# Successful Amputation of the Thigh at the Hip-Joint

**Published:** 1849-02

**Authors:** 


*Successful amputation of the Thigh at the Hip-Joint.*—M. Guersant operated, on the 28th December, 1847, for a cancerous affection of the femur by disarticulation at the hip-joint. The child, aged 5, was very much reduced. He was put under the influence of chloroform—insensibility was complete in two minutes. The operation lasted only two minutes. When the ligature was being placed on the vessels the child became pale, a little foam came from the mouth, the eyes were turned up, and the pulse at the wrist disappeared. This state of syncope was dissipated by means of active ventilation, and the introduction of a few spoonfuls of wine into the stomach—and the child began to cry, much to the relief of the surgeon. Twenty two days after the operation the child was as well as possible.—*Prov. Med. Surg. Jour. from Journ de Méd et de Chir.*, Fev. 1848.

